# Prolactin Variants in Human Pituitaries and Pituitary Adenomas Identified With Two-Dimensional Gel Electrophoresis and Mass Spectrometry

**DOI:** 10.3389/fendo.2018.00468

**Published:** 2018-08-28

**Authors:** Shehua Qian, Yongmei Yang, Na Li, Tingting Cheng, Xiaowei Wang, Jianping Liu, Xuejun Li, Dominic M. Desiderio, Xianquan Zhan

**Affiliations:** ^1^Key Laboratory of Cancer Proteomics of Chinese Ministry of Health, Xiangya Hospital, Central South University, Changsha, China; ^2^Hunan Engineering Laboratory for Structural Biology and Drug Design, Xiangya Hospital, Central South University, Changsha, China; ^3^State Local Joint Engineering Laboratory for Anticancer Drugs, Xiangya Hospital, Central South University, Changsha, China; ^4^Geriatric Department of Cadre's Ward, Baoji Traditional Chinese Medicine Hospital, Baoji, China; ^5^Bio-Analytical Chemistry Research Laboratory, Modern Analytical Testing Center, Central South University, Changsha, China; ^6^Department of Neurosurgery, Xiangya Hospital, Central South University, Changsha, China; ^7^The Charles B. Stout Neuroscience Mass Spectrometry Laboratory, Department of Neurology, College of Medicine, University of Tennessee Health Science Center, Memphis, TN, United States; ^8^The Laboratory of Medical Genetics, Central South University, Changsha, China

**Keywords:** prolactin variants, human pituitary, post-translational modifications, mass spectrometry, variant pattern

## Abstract

Human prolactin (hPRL) plays multiple roles in growth, metabolism, development, reproduction, and immunoregulation, which is an important protein synthesized in a pituitary. Two-dimensional gel electrophoresis (2DE) is an effective method in identity of protein variants for in-depth insight into functions of that protein. 2DE, 2DE-based PRL-immunoblot, mass spectrometry, and bioinformatics were used to analyze hPRL variants in human normal (control; *n* = 8) pituitaries and in five subtypes of pituitary adenomas [NF^−^ (*n* = 3)-, FSH^+^ (*n* = 3)-, LH^+^ (*n* = 3)-, FSH^+^/LH^+^ (*n* = 3)-, and PRL^+^ (*n* = 3)-adenomas]. Six hPRL variants were identified with different isoelectric point (*p*I)-relative molecular mass (*M*_*r*_) distribution on a 2DE pattern, including variants V1 (*p*I 6.1; 26.0 kDa), V2 (*p*I 6.3; 26.4 kDa), V3 (*p*I 6.3; 27.9 kDa), V4 (*p*I 6.5; 26.1 kDa), V5 (*p*I 6.8; 25.9 kDa), and V6 (*p*I 6.7; 25.9 kDa). Compared to controls, except for variants V2-V6 in PRL-adenomas, V2 in FSH^+^-adenomas, and V3 in NF^−^-adenomas, the other PRL variants were significantly downregulated in each subtype of pituitary adenomas. Moreover, the pattern of those six PRL variants was significantly different among five subtypes of pituitary adenomas relative to control pituitaries. Different hPRL variants might be involved in different types of PRL receptor-signaling pathways in a given condition. Those findings clearly revealed the existence of six hPRL variants in human pituitaries, and the pattern changes of six hPRL variants among different subtypes of pituitary adenomas, which provide novel clues to further study the functions, and mechanisms of action, of hPRL in human pituitary and in PRL-related diseases, and the potential clinical value in pituitary adenomas.

## Introduction

Prolactin (PRL) is a four long α-helix protein hormone, which was discovered in mammals in the 1930s by Oscar Riddle, and in humans in the 1970s by Friesen et al. (1). The PRL-encoded gene is located on chromosome 6 in the human, and consists of five exons and four introns (2). The hPRL cDNA consists of 914 nucleotides and includes a 681-nucleotide open-reading frame that encodes the PRL prohormone with 227 amino acids (positions 1–227), 25.9 kDa, which contains a signal peptide in amino acid positions 1-28 (Table 1). Mature hPRL contains 199 amino acids (positions 29–227), 22.9 kDa, which removed the signal peptides (positions 1–28). PRL was originally named because of the fact that it promotes lactation in rabbit (3). PRL is a polypeptide hormone with complex function, and is synthesized and secreted in the anterior pituitary gland, but also in other tissues and organs, such as skin, prostate, and the immune system. Pituitary PRL secretion is regulated by a series of factors that are derived from the external environment and internal milieu. In mammals, hypothalamic regulation of pituitary PRL secretion is largely inhibitory. The physiological stimuli include suckling, stress, and increased levels of ovarian steroids; primary estrogen can elevate pituitary PRL secretion. PRL is produced in autocrine/paracrine and endocrine systems. After secretion, PRL transports to the target tissues mammary gland, prostate, liver, and ovary via the blood circulation to bind to two different types of short or long PRL receptors (PRLRs) to activate signal pathways, which include JaK2 activation, Ras-Raf-MAPK pathway, modulatory pathways, PI3K and downstream pathways, and stats (4). PRLs that interact with different short or long PRLRs must be different PRL variants.

**Table 1:** The amino acid sequence of hPRL prohormone (Swiss-Prot No.: P01236; position 1–227; 227 amino acids long, and 25.9 kDa), and mature PRL (position 29-227; 199 amino acids long, 22.9 kDa).

10	20	30	40	50
**MNIKGSPWKG**	**SLLLLLVSNL**	**LLCQSVAP**LP	ICPGGAARCQ	VTLRDLFDRA
60	70	80	90	100
VVLSHYIHNL	SSEMFSEFDK	RYTHGRGFIT	KAINSCHTSS	LATPEDKEQA
110	120	130	140	150
QQMNQKDFLS	LIVSILRSWN	EPLYHLVTEV	RGMQEAPEAI	LSKAVEIEEQ
160	170	180	190	200
TKRLLEGMEL	IVSQVHPETK	ENEIYPVWSG	LPSLQMADEE	SRLSAYYNLL
210	220			
HCLRRDSHKI	DNYLKLLKCR	IIHNNNC		

*Signal peptide is position 1–28 in the underlined bold letters*.

Protein variants are mainly due to alternative splicing, post-translational modifications (PTMs), translocation, re-distribution, and spatial conformation alteration (5). Normal hPRL is 25.9 kDa with 227 amino acid residues for PRL prohormone in the pituitary gland or 22.9 kDa with 199 amino acid residues for mature PRL secreted into body fluid. However, PRL variants have been found in many mammals, which are derived from proteolytic cleavage, alternative splicing, and other PTMs such as phosphorylation, glycosylation, and polymerization in amino acid residues to result in changes in their *p*I and *M*_*r*_. Further studies found that the main source of PRL variants in mammals is not alternative splicing, but cleavage of PRL, and those variants were 14-, 16-, and 22-kDa (6). Liu et al. found one variant of hPRL with a sperm-penetration assay to be relative to breast cancer (7). Sohm et al. discovered two variants of PRL in the tilapia species Oreochromis niloticus and in fish (8). Although PRL is involved in osmotic regulation, PRL variants contribute to an osmotic adjustment disorder. Bollengier et al. indicated four variants of rat PRL in the pituitary cell with two-dimensional gel electrophoresis (2DGE), and also found that those variants were derived from different PTMs (9). Most of publications are involved in the studies of non-human PRL variants with identity of only a few non-human PRL variants (10–12). Some studies also found hPRL variants (*n* = 3–4) (13). However, until now 2DGE has not been used to study the hPRL variants.

2DGE is an effective method to separate proteins according to different *p*I values in the isoelectric focusing (IEF) direction, and different *M*_*r*_ values in the sodium dodecyl sulfate-polyacrylamide gel electrophoresis (SDS-PAGE) direction (14). *p*I and *M*_*r*_ are the basic properties of a protein variant. 2DGE-based Western blotting coupled with antibody of a given protein can also be used to detect the variants of that protein. Therefore, 2DGE is able to array PRL variants with different *p*I and *M*_*r*_. Mass spectrometry (MS) is an effect method to characterize the isolated PRL variants and identify PTMs with an analysis of amino acid sequence and determination of PTM sites. These types of MS have been used to study the 2DGE-separated pituitary proteins, including matrix-assisted laser desorption/ionization time-of-flight MS (MALDI-TOF MS) peptide fingerprint (PMF), MALDI-TOF-TOF MS and tandem mass spectrometry (MS/MS), and liquid chromatography-electrospray ionization-quadruple-ion trap MS (LC-ESI-Q-IT MS) MS/MS analysis (15–18).

This present study used 2DGE, 2DGE-based Western blotting, and MS to detect and identify hPRL variants in human pituitaries and differentially expressed profiles of hPRL variants among different subtypes of pituitary adenomas relative to controls, which will provide novel clues to further study the functions, and mechanisms of action, of hPRL in human pituitary and in PRL-related diseases, and the potential clinical value in pituitary adenomas.

## Materials and methods

### Tissue samples

Human control pituitary glands were post-mortem tissues obtained from the National Disease Research Interchange (*n* = 1) and the Memphis Regional Medical Center (*n* = 7), which were approved by University of Tennessee Health Science Center (UTHSC) Internal Review Board (IRB). Human pituitary adenoma tissues were obtained from the Emory University Hospital, which were approved by Emory University Hospital IRB, and Department of Neurosurgery of Xiangya Hospital, which were approved by the Xiangya Hospital Medical Ethics Committee of Central South University, China. Consent was obtained from each patient or the family of control pituitary subject after full explanation of the purpose and nature of all procedures used. All tissues were removed, frozen immediately in liquid nitrogen, and stored at −80°C until processing. The clinical information of pituitary adenoma and control samples is summarized in Table 2.

**Table 2:** Clinical information of human pituitaries and pituitary adenomas.

**Groups**	**Samples ID**	**Sex/Age**	**Clinical information**	**Immuno-histochemistry**
Control pituitary	C4	M/45	White, Drowning. Blood alcohol = 3.1 g/L; no other drugs detected. Blood: HepBb (+), HepC (+), HIV (–)	DNT
	C2	M/27	Black, none	DNT
	C3	F/40	White, Multiple toxic compounds. Blood: HepB (+), HepC (+), HIV (–)	DNT
	C5	M/36	White, Multiple toxic materials. Blood alcohol = 0.5g/L. Blood: HepB (+), HepC (–), HIV (–)	DNT
	C7	F/34	Black, Gunshot wound to chest. Blood alcohol = 0.3g/L; no drugs. Blood: HepB (+), HepC (–), HIV (–)	DNT
	C8	F	White, 15 h gunshot wound to head. No drugs or alcohol. Blood: HepB (–), HepC (–), HIV (–)	DNT
	C9	M/55	White, 12 h gunshot wound to chest. No alcohol or drugs. Blood: HepB (–), HepC (–), HIV (–)	DNT
	C10	F/47	White, smoke inhalation. No drugs or alcohol. Numerous amylacea present in brain. Early autolytic changes to brain. Blood: HepB (–), HepC (+), HIV (–)	DNT
Prolactinoma (PRL-PA)	T237	M/36	Prolactinoma, 1.918ng/ml, 2.0 × 2.1 × 2.5 cm	PRL3+
	T192	M/41	Prolactinoma,1.176ng/ml, 3 × 2.5 × 2.0 cm	PRL3+
	T131	F/52	Prolactinoma,359ng/ml, 2.5 × 3.5 × 2.8 cm	PRL3+
	T87	M/48	Prolactinoma with calcilication	PRL+
	T914933	F/45	Prolactinoma, with active cell growth, invasive tumor	PRL++
NF-NFPA	T219	M/68	Non-functional, 1.9 × 2.3 × 2.2 cm, invasion of the right cavernous sinus	Neg.
	T164	M/35	Non-functional, visual loss, 3 × 3.5 × 4 cm. Partial hypopituitarism	Neg.
	T217	M/39	Non-functional	Neg.
LH-NFPA	T208	F/47	Non-functional, 2 × 2 × 2 cm	LH 1–2+
	T204	M/47	Non-functional	LH 3+
	T237	F/40	Non-functional, right cavernous sinus extension	LH 2+
LH/FSH-NFPA	T65	F/54	Non-functional, 4 × 4 × 4 cm, cavernous sinus invasion	LH 2+, FSH 1+
	T138	M/60	Non-functional, 2.9 × 3.1 × 3.5 cm	LH 2+, FSH 2+
	T185	M/66	Non-functional, 2.8 ×, 2 × 2.4 cm. Bilateral cavernous sinus invasion	LH 2-3+, FSH 2-3+
FSH-NFPA	T57	F/59	Non-functional, 2 × 3 cm	FSH 1+
	T89	M/62	Non-functional, 2 × 2.3 × 2.3 cm	FSH 2+
	T77	M/67	Non-functional, 2 × 2.2 × 2.4 cm, questionable cavernous sinus	FSH 2+

*Neg, Immunohistochemical stains for ACTH, LH, FSH, PRL, GH, and TSH were negative. LH^+^, nonfunctional pituitary adenoma that expressed leuteinizing hormone, or lutropin; FSH^+^, nonfunctional pituitary adenoma that expressed follicle-stimulating hormone, or follitropin; FSH^+^ and LH^+^, nonfunctional pituitary adenoma that expressed both follicle-stimulating hormone and leuteinizing hormone. Adenomas were graded blindly by a neuropathologist (from 0 to 4) for the intensity of staining for each peptide hormone. NFPA, nonfunctional pituitary adenoma. DNT, Do not know*.

### Protein extraction

Tissue processing and protein extraction of pituitary control and adenoma tissues have been previously described (17, 19). Briefly, each tissue sample (~600 mg) was washed with 0.9% NaCl (3 ml, 5×) to thoroughly remove blood, and was finely ground in liquid nitrogen. A volume (5 ml) of protein extraction buffer that contained 2 mol/L thiourea, 7 mol/L urea, 40 g/L CHAPS, 100 mmol/L dithiothreitol (DTT), 5 mol/L immobilized pH gradient (IPG) buffer pH 3-10 NL, and a trace of bromphenol blue was added, and the mixture was thoroughly mixed. The mixture was vortexed (2 h) on ice and was centrifuged (15,000 × g, 15 min, 4°C). The supernatant was collected, and its protein concentration was determined with a Bio-Rad 2D Quant kit (Bio-Rad). The supernatant was the “protein sample solution.”

### 2DGE and western blot

#### First dimension—IEF

IEF was performed with precast IPG strips (pH 3-10 NL, 180 × 3 × 0.5 mm) and 18 cm IPG strip holder on an IPGphor instrument (GE Healthcare). Before IEF, an aliquot (350 μl) of protein sample solution was loaded onto an IPG strips. The IPG strip was rehydrated about 18 h. IEF was performed at 25°C with the following parameters: a gradient 250 V and 1 h for 125 Vh, a gradient at 1000 V and 1 h for 500 Vh, a gradient at 8000 V and 1 h for 4000 Vh, a step-and-hold at 8000 h and 4 h for 32000 Vh, a step-and-hold at 500 V and 0.5 h for 250 Vh to achieve a total of 36,875 Vh and ~7.5 h analysis. The IPG strip was removed and laid on its plastic back to blot off mineral oil. After IEF, IPG strips were processed with SDS-PAGE.

#### Second dimension—SDS-PAGE

After IEF, an Ettan DALT II system (GE Healthcare, up to 12 gels at a time) was used. An Ettan DALTsix multiple casters (GE Healthcare) was used to cast the 12% PAGE resolving gel (250 × 215 × 1.0 mm). The resolving-gel solution was made by mixing 75 ml of 1.5 mol/L Tris-HCl pH 8.8, 90 ml of 400 g/L acrylamide/bis-acrylamide (29:1 = weight:weight, cross-linking ratio = 3.3%), 3 ml of 10% ammonium persulfate, 150 ml deionized distilled water (ddH_2_O), and 50 μl of tetramethylethylenediamine. The IPG strip was equilibrated in 15 ml of reducing equilibration buffer (15 min) that consisted of a trace of bromphenol blue, 375 mmol/L Tris-HCl pH 8.8, 2% w/v SDS, and 20% v/v glycerol. The IPG strip was equilibrated in 15 ml of alkylation equilibrium solution (15 min) that consisted of 2.5% w/v iodoacetamide instead of 2% w/v DTT. A boiled solution that consisted of 1% w/v agarose solution was used to seal the equilibrated IPG strip onto the top of the resolving gel. 2DGE was performed in 25 L of Tris-glycine-SDS electrophoresis buffer that consisted of 192 mmol/L glycine, 25 mmol/L Tris-base, and 0.1 % w/v SDS at 25°C with a constant voltage (250 V, 360 min).

#### 2DGE-based western blot with anti-hPRL antibody

After electrophoresis, the 2D gel was removed, and proteins in the 2D gel were transferred to a polyvinylidene fluoride (PVDF) membrane (0.8 mA/cm^2^ for 1 h 20 min) with an Amersham Pharmacia Biotech Nova Blot semi-dry transfer instrument. The proteins on the PVDF membrane were blocked for 1 h at room temperature with a solution (100 ml) of 0.3% bovine serum albumin/phosphate-buffered saline (BSA/PBS) with 0.2% Tween 20 and 0.1% sodium azide (PBST). The proteins on the PVDF membrane were incubated for 1 h at room temperature with rabbit anti-hPRL antibodies that were diluted (v:v = 1:1000) in a 0.3% BSA/PBST solution. After incubation with the primary antibody, the PVDF membrane was washed with 200 ml PBST solution (15 min × 4) and rinsed twice with ddH_2_O. The secondary antibody, goat anti-rabbit alkaline phosphtase-conjugated IgG was diluted (v:v = 1:4000) in a 0.3% BSA/PBST solution. The solution was added to the blots for 1 h at room temperature. The membrane was washed with 200 ml PBST solution (15 min × 4), and proteins were visualized with 5-bromo-4-chloro-3-indolyl phosphate.

#### Protein staining and image analysis

All 2DGE-separated proteins were visualized with silver-staining (20). The silver-stained 2D gel was digitized and analyzed with Discovery Series PDQuest 2D Gel Analysis software (15, 21).

### MS analysis and database searching

#### MALDI-TOF MS

MALDI-TOF MS was used to analyze each protein that was digested with trypsin. Experiments were performed on a Perseptive Biosystems MALDI-TOF Volyager DE-RP MS (Framingham, MA, USA). The parameters of the instrument were described (15). The protonated molecule ion [M+H]^+^ was produced with MALDI-TOF MS. Data-processing software (DataExplore) was used to obtain accurate masses. Blank-gel experiments were conducted simultaneously to remove masses from known contaminants (usually keratin), matrix, trypsin, and other unknown contaminants. Each protein was identified by using the data obtained from MALDI-TOF MS PMF by searching the Swiss-Prot database 091215 (513877 sequences; 180750753 residues; January 19, 2015) with PeptIdent software.

#### LC-ESI-Q-IT MS

Proteins in 2D gel-spots that corresponded to each positive Western blot were excised and digested in the gel with trypsin. According to the manufacturer's method recommendations, the mixture of tryptic peptides was purified with a ZipTipC18 microcolumn. Purified tryptic peptides were analyzed with an LCQ^Deca^ mass spectrometer (Thermo-Finnigan, San Jose, CA, USA). The instrument parameters were: electron multiplier-900 V, ESI voltage 2.0 kV, and capillary probe temperature 110°C. The detailed experimental steps have been described (15). Data from LC-ESI-Q-IT MS were used to identify the protein with Swiss-Prot database.

#### MALDI-TOF-TOF MS

Proteins in the 2D gel-spots that corresponded to the positive Western blot were excised and digested in the gel with trypsin. For Perspective Biosystems MALDI-TOF-TOF MS analysis, the tryptic peptide extraction was eluted directly from a liquid chromatography microcolumn onto a MALDI plate with a matrix that contained 3 mg/ml saturated α-cyano-4-hydroxycinnamic acid solution. The purified tryptic peptide mixture was analyzed (*n* = 5). Tryptic peptides were analyzed with MALDI-TOF-TOF MS that operated in the reflective mode at an acceleration voltage of 25 kV over *m/z* 800-4000. Precursor ions close to the theoretical *m/z* were selected for TOF-TOF [UltraFlex III MALDI-TOF-TOF (Bruker Daltonics)] MS analysis. After automatic analysis, any remaining unidentified ions were manually analyzed. In the results obtained from MS, data compared to the theoretical values from the database were used to determine whether these peptides had undergone PTMs.

### Bioinformatics analysis

NetPhos 3.1 Server (http://www.cbs.dtu.dk/services/NetPhos) (22, 23), NetNGlyc 1.0 Server (http://www.cbs.dtu.dk/services/NetNGlyc) (24), and NetOGlyc 4.0 Server (http://www.cbs.dtu.dk/services/NetOGlyc) (25) were used to predict phosphorylation sites, N-glycosylation sites, and O-glycosylation sites in the hPRL in human pituitaries, respectively.

### Statistical analysis

The Chi-square test included in SPSS 22 software was used to analyze the difference in proportional ratio of PRL variants among five subtypes of pituitary adenomas, with a significance level of *p* = 0.05.

## Results

### 2DGE pattern of six human pituitary PRL variants and their differential expression changes among different subtypes of pituitary adenomas

**Figure 1:**
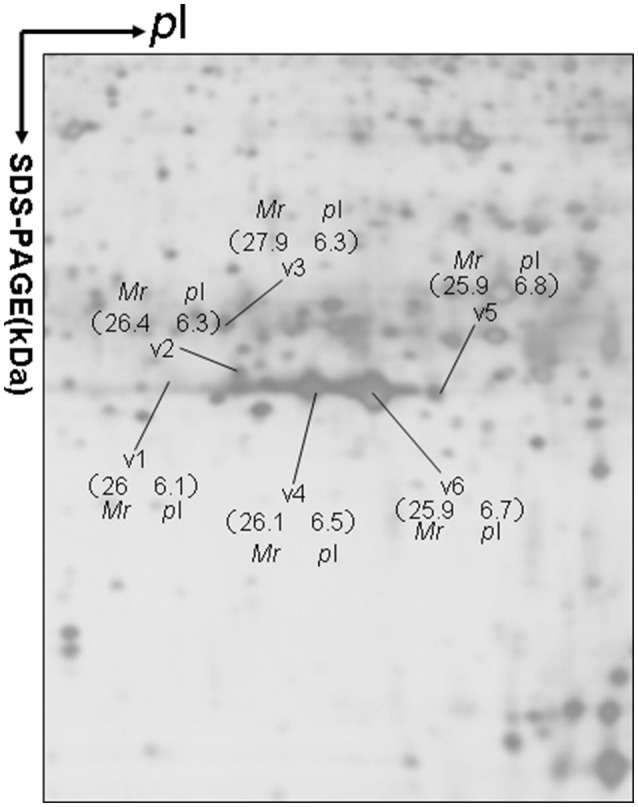
A representative 2DGE image (master gel image from a control) between a comparison between 8 control tissues and 16 human pituitary adenoma tissues. IEF was done with an 18-cm IPG strip (pH 3–10, nonlinear), and vertical SDS-PAGE was done with a 12% polyacrylamide gel.

Approximately 1,200 protein spots were detected in each silver-stained 2D gel. Six protein spots were found to contain PRL (Swiss-Prot No. P01236) (Figure 1). Six 2D gel spots were MS-identified to contain hPRL with a different *p*I-*Mr* pattern, including hPRL variants v1 (*p*I 6.1; 26.0 kDa), v2 (*p*I 6.3; 26.4 kDa), v3 (*p*I 6.3; 27.9 kDa), v4 (*p*I 6.5; 26.1 kDa), v5 (*p*I 6.8; 25.9 kDa), and v6 (*p*I 6.7; 25.9 kDa).

**Figure 2:**
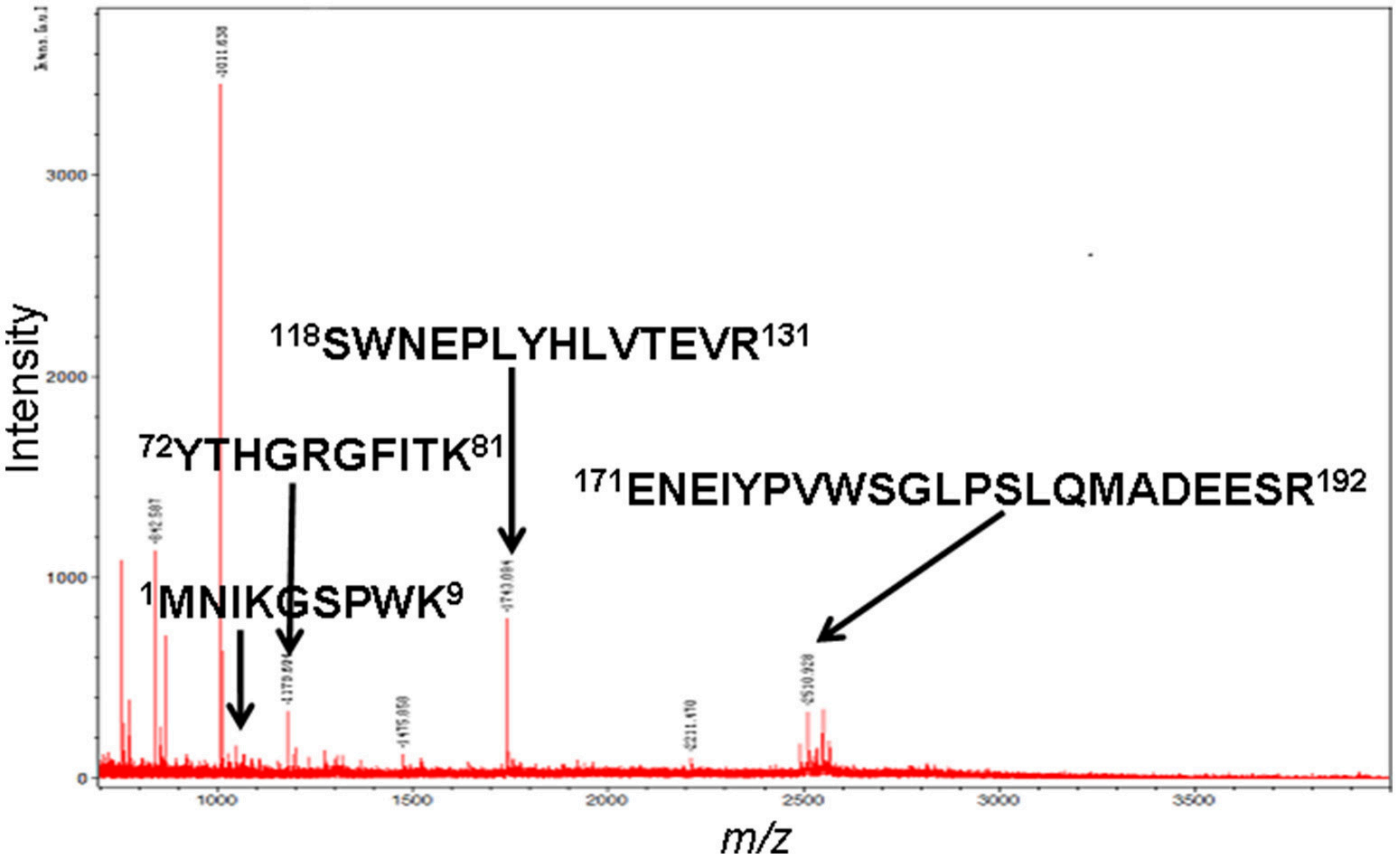
MS spectrum of hPRL that was contained in spot v6.

**Figure 3:**
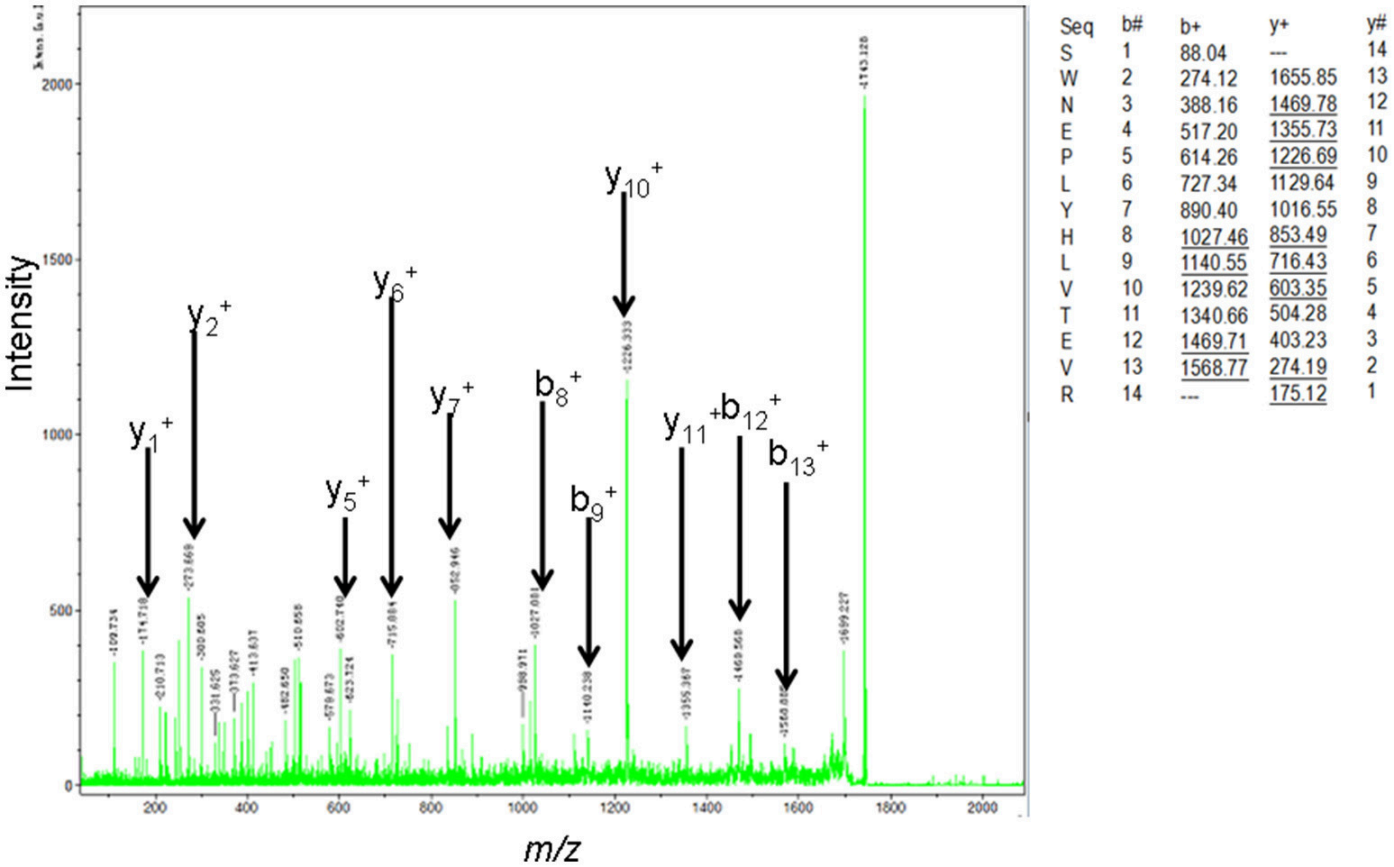
MS/MS spectrum of the tryptic peptide ^118^SWNEPLYHLVTEVR^131^ that was derived from hPRL in spot v6. The upper right corner is the theoretically calculated b- and y-ions for this analyzed tryptic peptide. The corresponding observed b- and y-ions were labeled in the MS/MS spectrum.

The MS-identification of hPRL in spot v6 will be used here as a representative example. The tryptic peptides from spot v6 were analyzed with MALDI-TOF MS or MALDI-TOT-TOF MS. The PMF data were obtained from spot V6 (Figure 2), and four peptides were significantly matched to hPRL (P01236) in the Swiss-Prot human database. Moreover, the tryptic peptides from spot V6 were also analyzed with MALDI-TOF-TOF MS/MS or LC-ESI-Q-IT MS/MS. Three peptides from spot V6 were sequenced with MS/MS data, including ^72^YTHGRGFITK^81^, ^118^SWNEPLYHLVTEVR^131^, and ^171^ENEIYPVWSGLPSLQMADEESR^192^, to significantly match with hPRL (P01236) in the Swiss-Prot human database. The MS/MS spectrum of the tryptic peptide ^118^SWNEPLYHLVTEVR^131^ contained a robust product-ion series that included b8, b9, b12, b13, y1, y2, y5, y6, y7, y10, y11, and y12 ions (Figure 3). Similarly, the hPRL in the other spots v1-v5 were identified (Figure 1 and Table 3).

**Table 3:** Prolactin variants changed in different subtypes of pituitary adenomas relative to control pituitaries.

**Variant No**.	**SSP No**.	**Protein description**	**Swiss-prot No**.	***p*I**	***M*_r_**	**Ratio (NF^−^: Con)**	**Ratio (FSH^+^/LH^+^: Con)**	**Ratio (FSH^+^: Con)**	**Ratio (LH^+^: Con)**	**Ratio (PRL: Con)**
V1	4106	Chain1:prolactin	P01236	6.1	26.0	−8.3	−99.9	−46.2	−12.6	−3.4
V2	4215^a)^	Prolactin precursor	P01236	6.3	26.4	−4.9	−3.8	1	−4.1	1
V3	4216	Chain1:prolactin	P01236	6.3	27.9	1	−12.3	−14.6	−26.2	1
V4	5114^b)^	Chain1:prolactin	P01236	6.5	26.1	−100	−19.0	−17.6	−20.1	1
V5	6109	Chain1:prolactin	P01236	6.8	25.9	−100	−19.7	−100	−36.7	1
V6	6119	Chain1:prolactin	P01236	6.7	25.9	−100	−32.6	−11.3	−33.6	1

a) characterized with LC-ESI MS/MS;

b) characterized with LC-ESI-MS/MS and MALDI-TOF PMF; all other proteins were characterized with MALDI-TOF PMF. LH^+^, NF that expressed leuteinizing hormone, or lutropin; FSH^+^, NF that expressed follicle-stimulating hormone, or follitropin; FSH^+^ and LH^+^, NF that expressed both follicle-stimulating hormone and leuteinizing hormone; NF-, NF that had negative immunohistochemical stains for ACTH, FSH, GH, LH, prolactin, and TSH. Each adenoma was graded blindly by a neuropathologist from 0 to 4 for intensity of staining for each peptide hormone. Con, control; -, decreased relative to controls;−100, lost relative to controls; 1, no change relative to controls; M_r_, kDa

**Figure 4:**
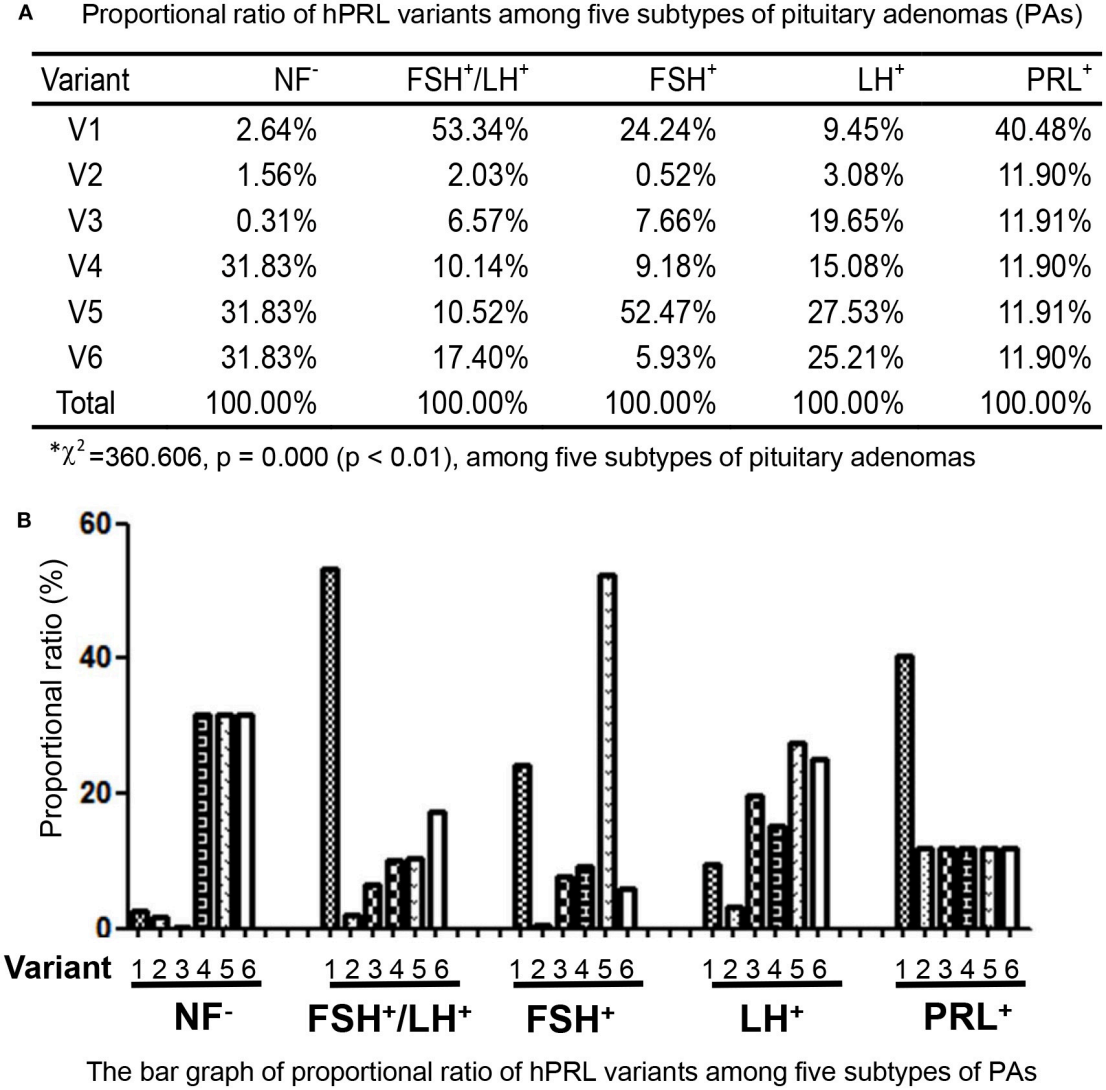
Comparison of the proportional ratio of PRL variants among different subtypes of pituitary adenomas.

In the non-hormone expressed nonfunctional pituitary adenoma (NF-NFPA) group relative to controls (Table 3), hPRL was downregulated by 8.3-fold in spot v1 and 4.9-fold in spot v2, was not changed in spot v3, and was lost in spots v4, v5, and v6. In the leuteinizing hormone (LH)-positive NFPAs relative to controls, hPRL was downregualted by 12.6-fold in spot v1, 4.1-fold in spot v2, 26.2-fold in spot v3, 20.1-fold in spot v4, 36.7-fold in spot v5, and 33.6-fold in spot v6. In the follicle-stimulating hormone (FSH)-positive NFPAs relative to controls, hPRL was not changed in spot v2, lost in spot v5, and was downregulated by 46.2-fold in spot v1, 14.6-fold in spot v3, 17.6-fold in spot v4, and 11.3-fold in spot v6. In FSH/LH-positive NFPAs relative to controls, hPRL in all spots was downregulated by 99.9-, 3.8-, 12.3-, 19.0-, 19.7-, and 32.6-fold, respectively. However, for prolactinomas (PRL-adenomas) relative to controls, hPRL was downregulated in only spot v1 by 3.4-fold, and not changed in the other spots. Moreover, the overall proportional ratio of six PRL variants was significantly different among the five subtypes of pituitary adenomas (NF^−^, FSH^+^-, LH^+^-, FSH^+^/LH^+^-, and PRL^+^-adenomas) analyzed here with the Chi-square test performed with SPSS 22 software (*p* < 0.05) (Figure 4). The Chi-square tests carried out between every two subtypes of pituitary adenomas indicated that the proportional ratio of six hPRL variants was significantly different between every two subtypes of pituitary adenomas (*p* < 0.05), except for no significant difference between subtypes FSH^+^/LH^+^ and PRL^+^, and between subtypes FSH^+^ and PRL^+^.

### Validation of hPRL variants with 2DGE-based PRL immunoaffinity blot

**Figure 5:**
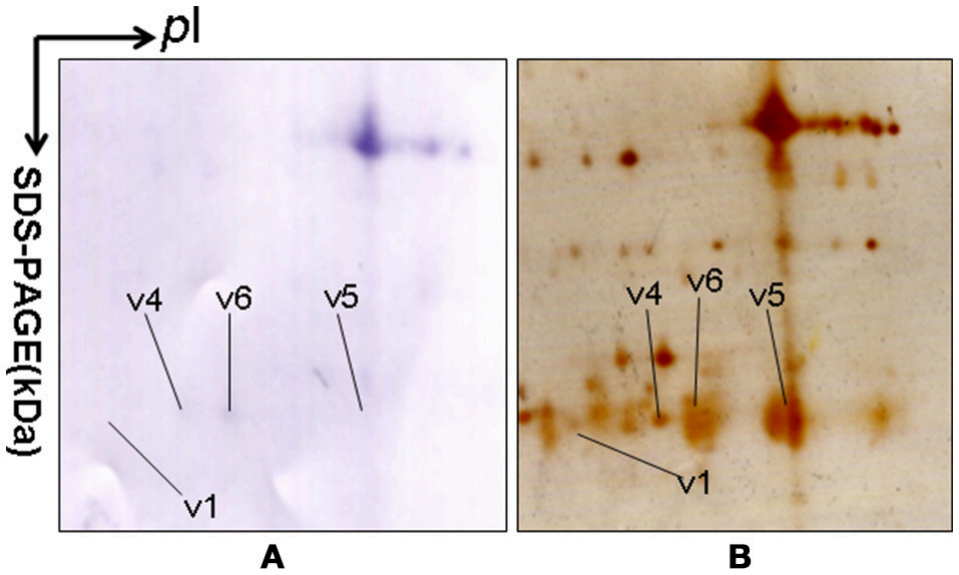
2DGE-based Western blot confirmed hPRL variants in pituitary tissues. IEF was done with an 18-cm IPG strip (pH 3-10, nonlinear), and vertical SDS-PAGE was done with a 12% polyacrylamide gel. The primary antibody was rabbit anti-hPRL antibody. The second antibody was goat anti-rabbit alkaline phosphtase-conjugated IgG. **(A)** Western blot image of anti-hPRL proteins (rabbit anti-hPRL antibodies + goat anti-rabbit alkaline phosphtase-conjugated IgG). **(B)** Silver-stained image on a 2D gel after transfer of protein to a PVDF membrane.

2DGE-based Western blot coupled with anti-hPRL antibody and MS was an effective method to validate hPRL variants in human pituitaries. Four hPRL variants in human pituitary, including variants v1, v4, v5, and v6, were detected with an hPRL Western blot-immunopositive reaction (Figure 5). Furthermore, the protein in each immunopositive spot (v1, v4, v5, and v6) was identified as PRL (Swiss-Prot No. P01236) with MALDI-TOF-MS PMF data and MALDI-TOF-TOF MS/MS. The PMF data (calculated; observed) of those four validated hPRL variants are collected in Table 4. Two tryptic peptides were obtained in spot v1, including ^72^YTHGRGFITK^81^ with a [M+H]^+^ ion at *m/z* 1179.7 and ^118^SWNEPLYHLVTEVR^131^ with a [M+H]^+^ ion at *m/z* 1743.0. Two tryptic peptides that is the same as spot v1 were also identified in spot v4. Compared to spots v1 and v4, one more tryptic peptide ^171^ENEIYPVWSGLPSLQMADEESR^192^ with a [M+H]^+^ ion at *m/z* 2550.6 was identified in spot v6. The tryptic peptide ^118^SWNEPLYHLVTEVR^131^ of PRL in spot v6 was also analyzed with MALDI-TOF-TOF MS/MS. Those two tryptic peptides ^118^SWNEPLYHLVTEVR^131^ and ^171^ENEIYPVWSGLPSLQMADEESR^192^, were also identified at spot v5, with [M+H]^+^ ions at *m/z* 1743.0 and 2550.2, respectively.

**Table 4:** Tryptic peptides of prolactin identified with mass spectrometry.

**Variant No**.	**Tryptic peptide**	**Calculated [M+H]^+^ ion (m/z)**	**Observed [M+H]^+^ ion (*m/z*)**
V1	^72^YTHGRGFITK^81^	1178.6	1179.7
	^118^SWNEPLYHLVTEVR^131^	1741.9	1743.0
V4	^72^YTHGRGFITK^81^	1178.6	1179.7
	^118^SWNEPLYHLVTEVR^131^	1741.9	1743.0
V6	^72^YTHGRGFITK^81^	1178.6	1179.7
	^118^SWNEPLYHLVTEVR^131^	1741.9	1743.1
	^171^ENEIYPVWSGLPSLQMADEESR^192^	2549.2	2550.6
V5	^118^SWNEPLYHLVTEVR^131^	1741.9	1743.0
	^171^ENEIYPVWSGLPSLQMADEESR^192^	2549.2	2550.2

*The calculated [M+H]^+^ ion of each PRL tryptic peptide was obtained with the EXPASY-PeptideMass tool (https://web.expasy.org/peptide_mass/). The observed [M+H]^+^ ion in each MALDI-TOF PMF spectrum was compared to the calculated [M+H]^+^ ion to determine the status of PRL variant in each 2-D gel spot*.

2DGE-based Western blot coupled with anti-hPRL antibody and MS clearly confirmed hPRL variants that were contained in spots v1, v4, v5, and v6 (Figure 1 vs. Figure 5). However, the hPRL variants in spots v2 (*p*I 6.3; 26.4 kDa) and v3 (*p*I 6.3; 27.9 kDa) were not confirmed; that finding might be due to several factors: (i) the PRL antibody was a general antibody, not specific to a given variant, and (ii) the *M*_*r*_ of hPRL in the spots v2 and v3 was larger than the *M*_*r*_ (25.9 kDa) of hPRL, obviously some adducts were added to the hPRL, which could be result in its non-reaction with the hPRL antibody. Whether the hPRL was detected with an immunoblot or not the hPRL in spots v2 and v3 was unequivocally identified with MS and MS/MS (Figure 1). Therefore, hPRL in spots v2 and v3 is still recognized as the hPRL variants in human pituitaries.

### Determination of signal peptide (position 1-28) contained in each hPRL variant in human pituitaries

PRL in human Swiss-Prot database contains 227 amino acids (position 1–227) with a signal peptide (position 1-28) (Table 1), which is a prohormone of hPRL with a molecular weight of 25.9 kDa, whereas mature hPRL contains 199 amino acids (position 29–227) with a removal of the signal peptide (position 1–28), with a molecular weight of 22.9 kDa. Therefore, it is necessary to determine whether those identified six hPRL variants are derived from hPRL prohormone or mature hPRL. With the theoretical calculation using PeptideMass Cleavage software (Table 5), there are seven characteristic tryptic peptides (positions 1–4, 1–9, 1–38, 5–9, 5–38, 10–38, and 10–44) derived from hPRL prohormone (position 1–227) that can be used to determine the signal peptide (position 1–28) in the identified hPRL variants, and there are three characteristic tryptic peptides (positions 29–38, 29–44, and 29–49) derived from mature hPRL (position 29–227) that can be used to determine the removal of signal peptide (position 1–28) in the identified hPRL variants. Comprehensive analysis of all MS data and MS/MS data of hPRL in spots v1–v6, demonstrated that three characteristic tryptic peptides (positions 29–38, 29–44, and 29–49) were not found (Table 5), and that one characteristic tryptic peptide (position 1–9, MNIKGSPWK, [M+H]^+^ *m/z* 106.5608) was identified (Table 5 and Figure 2). Two small characteristic tryptic peptides (position 1-4, MNIK, [M+H]^+^ *m/z* 505.2803; position 5–9, GSPWK, [M+H]^+^ *m/z* 574.2983) were not detected because they were out of the range of MS acquisition (Figure 2). Moreover, from the 2DGE pattern of six hPRL variants (Figure 1), it is clear that variants v1, v4, v5, and v6 had the corresponding *M*_*r*_ of 26.0 kDa, 26.1 kDa, 25.9 kDa, and 25.9 kDa, respectively, which are very close to the molecular weight of hPRL prohormone 25.9 kDa; and that the variants v2 and v3 have the corresponding *M*_*r*_ of 26.4 kDa, and 27.9 kDa, which is a bit larger than the molecular weight of hPRL prohormone 25.9 kDa. Therefore, according to MS data and 2DGE pattern, the identified six hPRL variants in human pituitary tissue should be derived from hPRL prohormone (position 1–227), but not from mature hPRL (position 29–227).

**Table 5:** Characteristic tryptic peptides to determine signal peptide (position 1–28) within the identified hPRL variants.

**Calc. [M+H]^+^**	**Position**	**Characteristic tryptic peptide sequence**	**Observed [M+H]^+^**
505.2803	1–4	MNIK	–
1060.5608	1–9	MNIKGSPWK	+
3930.1893	1–38	MNIKGSPWKGSLLLLLVSNL LLCQSVAPLPICPGGAAR	–
574.2983	5–9	GSPWK	–
3443.9268	5–38	GSPWKGSLLLLLVSNLLLCQ SVAPLPICPGGAAR	–
2888.6463	10–38	GSLLLLLVSNLLLCQSVAPL PICPGGAAR	–
3589.0154	10–44	GSLLLLLVSNLLLCQSVAPL PICPGGAARCQVTLR	–
954.5189	29–38	LPICPGGAAR	–
1654.8879	29–44	LPICPGGAARCQVTLR	–
2301.1954	29–49	LPICPGGAARCQVTLRDLFD R	–

*+, this peptide ion was observed with mass spectrometry in each MS spectrum. -, this peptide was not observed with mass spectrometry*.

### Bioinformatic prediction of potential factors to result in hPRL variants

Alternative splicing and PTMs are two main factors to result in protein variants. From the analysis in the section determination of signal peptide (position 1–28) contained in each hPRL variant in human pituitaries, six identified hPRL variants were not from splicing and truncation. Thus, PTMs might be the main factor to result in six hPRL variants. In the 2DGE map of hPRL variants (Figure 1), variants v1, v4, v5, and v6 had the very similar *M*_*r*_, but with obviously measurably different p*I*. The deamidation of an asparagine (N) residue to aspartate (D) and of glutamine (Q) residue to glutamate (E) is an effect of protein aging, and is often observed in 2-D gels (26–29). Deamidation results in a series of spots with the same *M*_*r*_ values but measurably different *p*I values (30), with an increase of 1 Da in peptide mass and a decrease in the apparent p*I* from the uncharged amide to a negatively charged carboxylate anion at pH 7.4. Moreover, deamidation might be produced under the basic conditions for storing samples (30).

In the 2DGE map of hPRL variants (Figure 1), variants v2 and v3 had the same apparent p*I* but obviously measurably different *M*_*r*_, and their *M*_*r*_ 26.4 kDa and 27.9 kDa were a bit larger than hPRL prohormone (25.9 kDa). This phenomenon might arise from some PTMs with a relatively larger chemical group, such as glycosylation or phosphorylation. Glycosylation means that the protein contains one or more covalently linked carbohydrates of various types from monosaccharides to branched polysaccharides. Phosphorylation means that the protein is modified by the attachment of either a single phosphate group, or of a complex molecule such as 5'-phospho-DNA through a phosphate group at the targeted serine, threonine, or tyrosine residues. The annotation page of hPRL in the UniProt database (https://www.uniprot.org/uniprot/P01236); P01236, or PRL_HUMAN) clearly demonstrates the hPRL is a glycoprotein or phosphoprotein. Moreover, multiple phosphorylated sites at the serine (S), threonine (T), and tyrosine (Y) residues were significantly predicted by NetPhos 3.1 Server, including 14 pS sites, 5 pT sites, and 3 pY sites in the hPRL (Table 6). Ten significantly N-glycosylated sites were predicted by NetNGlyc 1.0 Server in the hPRL (Table 7), and six significantly O-glycosylated sites were predicted by NetOGlyc 4.0 Server in the hPRL (Table 8).

Table 6Prediction of phosphorylation sites in hPRL (position 1–227) with NetPhos 3.1 Server with a score more than 0.5.

**Table:** 

**Sequence**	**#**	**x**	**Context**	**Score**	**Kinase**	**Answer**
Sequence	6	S	NIKGSPWKG	0.779	unsp	YES
Sequence	11	S	PWKGSLLLL	0.848	PKA	YES
Sequence	18	S	LLLVSNLLL	0.523	cdc2	YES
Sequence	42	T	RCQVTLRDL	0.891	unsp	YES
Sequence	61	S	IHNLSSEMF	0.718	unsp	YES
Sequence	62	S	HNLSSEMFS	0.553	unsp	YES
Sequence	66	S	SEMFSEFDK	0.991	unsp	YES
Sequence	72	Y	FDKRYTHGR	0.503	INSR	YES
Sequence	73	T	DKRYTHGRG	0.557	unsp	YES
Sequence	90	S	CHTSSLATP	0.585	DNAPK	YES
Sequence	93	T	SSLATPEDK	0.737	unsp	YES
Sequence	110	S	KDFLSLIVS	0.507	PKA	YES
Sequence	118	S	SILRSWNEP	0.749	unsp	YES
Sequence	124	Y	NEPLYHLVT	0.956	unsp	YES
Sequence	142	S	EAILSKAVE	0.517	CKII	YES
Sequence	151	T	IEEQTKRLL	0.983	unsp	YES
Sequence	163	S	ELIVSQVHP	0.623	ATM	YES
Sequence	169	T	VHTEPKENE	0.541	CKII	YES
Sequence	175	Y	ENEIYPVWS	0.804	unsp	YES
Sequence	191	S	ADEESRLSA	0.576	cdc2	YES
Sequence	194	S	ESRLSAYYN	0.982	unsp	YES
Sequence	207	S	LRRDSHKID	0.993	unsp	YES

**Table:** 

MNIKGSPWKGSLLLLLVSNLLLCQSVAPLPICPGGAARCQVTLRDLFDRA		#		50	
VVLSHYIHNLSSEMFSEFDKRYTHGRGFITKAINSCHTSSVTLRDLFDRA		#		100	
QQMNQKDFLSLIVSILRSWNEPLYHLVTEVRGMQEAPEAILSKAVEIEEQ		#		150	
TKRLLEGMELIVSQVHPETKENEIYPVWSGLPSLQMADEESRLSAYYNLL		#		200	
HCLRRDSHKIDNYLKLLKCRIIHNNNC		#		250	
**. . .** . S **. .** . S **. . . .** . S **. . . . . . . . . . . . . . . . . . . . .** . T **. . . . . .** .		#		50	
**. . . . . . . .** . SS **.** . S **. . .** . YT **. . . . . . . . . . . . .** . S . T **. . . . . .** .		#		100	
**. . . . . . .** . S **. . . . .** . S **. . .** . Y **. . . . . . . . . . . . . . .** . S **. . . . . .** .		#		150	
**. . . . . . . . .** . S **. . .** . T **. . . .** . Y **. . . . . . . . . . . .** . S . S **. . . .** .		#		200	
**. . . .** . S **. . . . . . . . . . . . . . . . . .** .				

Table 7Prediction of N-glycosylation sites in hPRL (position 1–227) with NetNGlyc 1.0 Server with score more than 0.5.

**Table:** 

**MNIKGSPWKGSLLLLLVSNLLLCQSVAPLPICPGGAARCQVTLRDLFDRAVVLSHYIHNLSSEMFSEFDKRYTHGRGFIT**	**80**
**KAINSCHTSSVTLRDLFDRAQQMNQKDFLSLIVSILRSWNEPLYHLVTEVRGMQEAPEAILSKAVEIEEQTKRLLEGMEL IVSQVHPETKENEIYPVWSGLPSLQMADEESRLSAYYNLLHCLRRDSHKIDNYLKLLCRIHNNNC**	**160**
**. n . . . . . . . . . . . . . . . . n . . . . . . . . . . . . . . . . . . . . . . . . . . . . . . . . . . . . . . . N . . . . . . . . . . . . . . . . . . .** .	**80**
**. . . n . . . . . . . . . . . . . . n . . . . . . . . . . . . . . . n . . . . . . . . . . . . . . . . . . . . . . . . . . . . . . . . . . . . . .** .	**160**
**. . . . . . . . . . . n . . . . . . . . . . . . . . . . . . . . . . . . . n . . . . . . . . . . . . . n . . . . . . . . . n .**.

**Table:** 

**SeqName**	**Position**	**Potential**	**Jury Agreement**	**N-Glyc result**	
Sequence	2 NIKG	0.7530	(9/9)	+++	
Sequence	19 NLLL	0.7151	(9/9)	++	
Sequence	59 NLSS	0.7380	(9/9)	++	
Sequence	84 NSCH	0.7312	(8/9)	+	
Sequence	104 NQKD	0.6020	(7/9)	+	SEQUON
Sequence	120 NEPL	0.6051	(6/9)	+	ASN-XAA-SER/THR.
Sequence	172 NEIY	0.5346	(5/9)	+	
Sequence	198 NLLH	0.5642	(5/9)	+	
Sequence	212 NYLK	0.6726	(8/9)	+	
Sequence	224 NNNC	0.5146	(5/9)	+	
Sequence	225 NNC-	0.3576	(8/9)	–	
Sequence	226 NC–	0.3351	(9/9)	–	

*Asn-Xaa-Ser/Thr sequons in the sequence are highlighted in blue. Asparagines predicted to be N-glycosylated are highlighted in red*.

**Table 8:** Prediction of O-glycosylation sites in hPRL (position 1–227) with NetOGlyc 4.0 Server with score more than 0.5.

**#Seq name**	**Source**	**Feature**	**Start**	**End**	**Score**	**Strand**	**Frame**	**Comment**
SEQUENCE	netOGlyc-4.0.0.13	CARBOHYD	25	25	0.134588	·	·	
SEQUENCE	netOGlyc-4.0.0.13	CARBOHYD	42	42	0.190888	·	·	
SEQUENCE	netOGlyc-4.0.0.13	CARBOHYD	54	54	0.194926	·	·	
SEQUENCE	netOGlyc-4.0.0.13	CARBOHYD	66	66	0.176466	·	·	
SEQUENCE	netOGlyc-4.0.0.13	CARBOHYD	73	73	0.111052	·	·	
SEQUENCE	netOGlyc-4.0.0.13	CARBOHYD	80	80	**0.613645**	·	·	#POSITIVE
SEQUENCE	netOGlyc-4.0.0.13	CARBOHYD	85	85	**0.618483**	·	·	#POSITIVE
SEQUENCE	netOGlyc-4.0.0.13	CARBOHYD	88	88	**0.602886**	·	·	#POSITIVE
SEQUENCE	netOGlyc-4.0.0.13	CARBOHYD	89	89	**0.717093**	·	·	#POSITIVE
SEQUENCE	netOGlyc-4.0.0.13	CARBOHYD	90	90	**0.928857**	·	·	#POSITIVE
SEQUENCE	netOGlyc-4.0.0.13	CARBOHYD	93	93	**0.778272**	·	·	#POSITIVE
SEQUENCE	netOGlyc-4.0.0.13	CARBOHYD	128	128	0.181904	·	·	
SEQUENCE	netOGlyc-4.0.0.13	CARBOHYD	151	151	0.11243	·	·	
SEQUENCE	netOGlyc-4.0.0.13	CARBOHYD	163	163	0.424529	·	·	
SEQUENCE	netOGlyc-4.0.0.13	CARBOHYD	169	169	0.122664	·	·	
SEQUENCE	netOGlyc-4.0.0.13	CARBOHYD	179	179	0.380532	·	·	
SEQUENCE	netOGlyc-4.0.0.13	CARBOHYD	183	183	0.309982	·	·	
SEQUENCE	netOGlyc-4.0.0.13	CARBOHYD	191	191	0.1589	·	·	
SEQUENCE	netOGlyc-4.0.0.13	CARBOHYD	194	194	0.249957	·	·	

*The bold values mean statistical significantly positive results*.

## Discussion

### Biological functions of PRL in different physiological and pathological conditions

PRL plays an extremely important role in different vertebrate species and organ systems, such as growth, metabolism, development, reproduction, immunoregulation (31), behavior and brain (6), water and balance (32), stress response, and suppression of (33–35) and regulation of firing of oxytocin neurons (36, 37). As it is named, PRL exerts multiple effects that include development (mammogenesis) and stimulation of mammary gland growth, synthesis of milk (lactogenesis), and maintain the secretion of milk (galactopoiesis) on the mammary gland (38). Moreover, PRL affects reproduction of the mammalian species. During pregnancy, PRL concentrations are high. During pregnancy, low immunity might be associated with PRL. Decreased serum PRL might cause anxiety in the first trimester of pregnancy (39). PRL induces and maintains maternal behaviors in rabbit, rat, hamsters, and sheep (40). These maternal behaviors include cleaning, nesting, grouping, and nursing of the baby by the mother (41, 42). Aside from the effects of PRL on reproductive processes, PRL also plays a significant role to maintain homeostasis of the body's environment by development of blood vessels (angiogenesis), regulation of the immune systems, and osmotic balance (3). PRL is also involved in the synthesis and secretion of milk, and the major ions in milk are sodium, potassium, and chloride ions. The levels of chloride and sodium ions in milk are low, and potassium is high (43). PRL might maintain low levels of sodium in milk because it can promote sodium retention in the mammary gland.

Moreover, PRL is involved in some endocrine-related cancers. PRL plays a vital role in the regulation of the immune responses; it interferes with inhibiting apoptosis, induces B-cell tolerance, increases antibody secretion, enhances antigen presentation, and upregulates cytokine production (44). PRL has different functions in the immune system of healthy people, including Treg cell inhibitory factor and inhibition of cell secretion (45). PRL contributes to the inflammatory microenvironment by reducing the inhibitory effect of Treg cells on T cells (45, 46). The proteolytic fragments of native PRL inhibit angiogenesis. Anti-angiogenic activity is achieved by the interaction between the 16 kDa N-terminal fragment of PRL and a specific receptor (47). Under normal conditions, PRL can promote the growth and development of the mammary gland, and the 16 kDa fragment of PRL can inhibit angiogenesis. Dilatation and regression of blood vessels seriously affect the normal growth and degeneration of breast cancer. However, angiogenesis disorders are the characteristics of growth and metastasis of breast cancer. That is not given physiological significance. It seems possible that the 16-kDa fragment of PRL plays a pathological role to inhibit angiogenesis or a local inhibitor of tumorigenesis.

### Determination of the number of hPRL variants in human pituitaries

The pituitary proteome was separated with 2DGE, followed by MS identification. Six 2D gel spots were found to contain hPRL with MS-identification, which represents six hPRL variants in human pituitaries. Of them, four hPRL variants (v1, v4, v5, and v6) were confirmed with 2DGE-based Western blotting in combination with anti-hPRL antibodies and followed by MS identification. However, two hPRL variants (v2 and v3) was not detected with 2DGE-based Western blotting in combination with anti-hPRL antibodies, which is due to the factors: (i) anti-hPRL antibody was not a variant-specific antibody, and (ii) those two hPRL variants might have different properties including different PTMs and unknown factors to cause its no-reaction with anti-hPRL antibody. Anyway, this present study directly MS-identified the hPRL in six different 2D gel spot with different *p*I and *M*_*r*_. Therefore, the present study still used the six hPRL variants to study their differential expression profile among different subtypes of pituitary adenomas.

### Formation of hPRL variants in human pituitaries

There are many factors that might produce protein variants: (i) Estimates of DNA level variation. Major sources of human protein variation include the encoding of single nucleotide polymorphisms and mutations. (ii) The main source of RNA level variation. Alternative splicing is a key factor in transcriptome complexity and regulating complex human characteristics. (iii) Errors in translation. Errors in protein translation provide a very large potential source of proteome expansion, especially in stressed or aging cells. (iv) PTMs. Due to PTMs, the potential number of proteoforms can increase exponentially (48). PTMs might have a certain influence on the structure and function of the protein; for example, PTMs of α-synuclein are major regulators of their function, structure, degradation, and toxicity (49). PTMs of the FUS protein might affect its associated pathology and serve as a therapeutic target (50). Currently, different types of PTMs have been found in the human, including glycosylation, phosphorylation, acetylation, ubquitination, methylation, deamidation, and nitration (27, 28, 51). These factors could also produce hPRL variants.

The present study found that six PRL variants that are derived from the same PRL gene in human pituitaries were not due to alternative splicing, but rather to PTMs, including deamidation, glycosylation, and phosphorylation. The hPRL variants v1, v4, v5, and v6 on the 2DGE map had a very similar *M*_*r*_ but obviously different p*I*, which might be mainly due to deamidaton. The hPRL variants v2 and v3 with a slightly larger *M*_r_ relative to hPRL (position 1–227) might be mainly due to the glycosylation and phosphorylation. Therefore, PTMs might be the main reason to produce hPRL variants. Furthermore, we confirmed that the amino acid sequence of six identified hPRL variants in human pituitaries contained the signal peptide (position 1–28), which is the hPRL prohormone (position 1–227), but not the mature PRL (position 29–227).

### Potential molecular mechanisms of action of hPRL variants elucidated by signaling pathway networks

**Figure 6:**
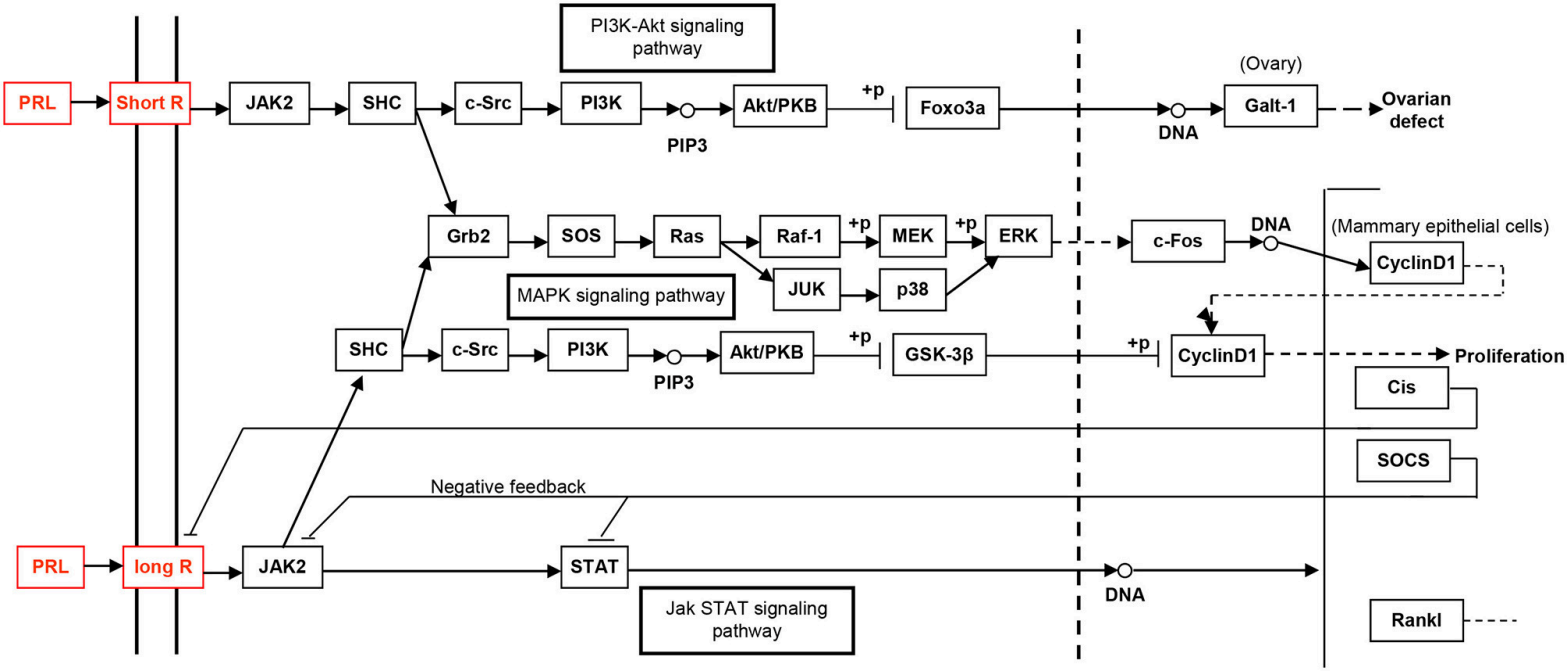
The PRL-driven signaling pathways via the short or long PRL receptors, which was achieved from KEGG pathway database.

The study of the hPRL signaling pathway network is helpful to understand its functions; explore molecular mechanism of PRL-related tumors such as pituitary adenomas, including prolactinomas; develop effective therapeutic drugs; and discover tumor biomarkers (52). Only after the interaction between PRL and its receptors can PRL play a corresponding function. For example, for the secretion of milk, the binding of PRL and PRL receptor (PRLR) does not activate cell membrane binding enzyme, but phospholipase A. The signaling pathway map of hPRL is complex (Figure 6). There are two types of PRLRs: long-PRLR and short-PRLR. PRL binding to different receptors can cause different physiological functions. The signaling pathways activated by PRL binding to different receptors are specific and exhibit crossover. PRL binding to short receptors activates the PI3K/Akt signaling pathway, whereas PRL binding to long receptors activates the JAK2/STAT signaling pathway. The cross-signaling pathway activated by the binding of PRL to long or short receptors is a MAPK signaling pathway that activates cell cycle regulators and thus affects cell proliferation. The different short or long PRLRs should bind different hPRL variants v1–v6 to activate different signaling pathways and produce different biological effects in different physiological and pathological conditions. Furthermore, the significantly differential expression of six hPRL variants (Table 3) might result in different signaling pathway alteration. The further studies on the association of different hPRL variants and PRLR signaling pathway might have important scientific merits and clinical significance.

## Strength and limitation

The present study clearly demonstrated the existence of six hPRL variants in human pituitaries with 2DGE and MS analyses, and its differential expression profile of six hPRL variants among different subtypes of pituitary adenomas; and confirmed that those six hPRL variants in human pituitary tissues were derived from hPRL prohormone with 227 amino acids (position 1–227), but not from mature hPRL with 199 amino acids (position 29–227).

However, mature PRL is secreted by prolactotroph cells concentrated on both sides of the posterior pituitary gland and enters the bloodstream after secretion. After blood transport, PRL will bind to its specific receptor before it can play a corresponding physiological role. PRL in pituitary tissue is difficult to detect with routine examinations, but PRL in blood is relatively easy to detect. Under normal circumstances, the level of PRL in the blood plays its role in a certain range. The abnormal secretion of PRL is related to many diseases, such as hyperprolactinemiaemia, tumor, and autoimmune diseases. Studies have shown that PRL has a direct or indirect relationship with many diseases. Whether these diseases are associated with serum PRL variants remains unknown. Therefore, it is necessary in our future studies to in-depth investigate the mechanisms and function of different PRL variants and the status of serum PRL variants. The present study provides the basis for us to in-depth study serum PRL variants and their functions.

## Conclusion

2DGE, 2DGE-based Western blot coupled with anti-hPRL antibody, MS, and bioinformatics were used to identify hPRL variants in human pituitaries, and their differential expression profiles among different pituitary adenomas. Six hPRL variants in human pituitaries were identified, including two hPRL variants that were not detected by Western blotting. The differential expression patterns of six hPRL variants were significantly different among five subtypes of pituitary adenomas (NF^−^, FSH^+^-, LH^+^-, FSH^+^/LH^+^-, and PRL^+^-adenomas). Moreover, six hPRL variants in human pituitaries derived from hPRL prohormone (position 1–227) with different PTMs such as deamidation, glycosylation, and phosphorylation, but not from mature hPRL (position 29–227). Those findings provide novel insights into the functions, and mechanisms of action, of hPRL in human pituitary and in PRL-related diseases, and into the potential clinical value in pituitary adenomas. Furthermore, the hPRL variants involved signaling pathways that might clarify the biological functions of different PRL variants and their potential clinical significance, and contribute to the development of drugs that block the PRL signaling pathway for clinical treatment.

## Author contributions

SQ analyzed data, prepared figures and tables, designed, and wrote the manuscript. YY collected tissue samples, performed clinical explanation, and carried out bioinformatics analysis and partial revision of manuscript. NL, TC, XW, and JL participated in experiments and partial data analysis. XL collected tissue samples and performed clinical diagnosis. DD participated in design, instructed, and critically revised manuscript. XZ conceived the concept, designed experiments, and manuscript, instructed experiments and data analysis, supervised results, coordinated, critically revised/wrote manuscript, and was responsible for its financial supports and the corresponding works. All authors approved the final manuscript.

### Conflict of interest statement

The authors declare that the research was conducted in the absence of any commercial or financial relationships that could be construed as a potential conflict of interest.

## Abbreviations

2DGE: two-dimensional gel electrophoresis
BSA: bovine serum albumin
ddH_2_O: deionized distilled water
DTT: dithiothreitol
ESI: electrospray ionization
FSH: follicle-stimulating hormone
hPRL: human prolactin
IEF: isoelectric focusing
IPG: immobilized pH gradient
LC: liquid chromatography
LH: leuteinizing hormone
MALDI: matrix-assisted laser desorption ionization
MS: Mass spectrometry
M_r_: relative molecular mass
PBS: phosphate-buffered saline
pI: isoelectric point
PRL: prolactin
PRLR: prolactin receptor
PTMs: post-translational modifications
PVDF: polyvinylidene fluoride
SDS-PAGE: sodium dodecyl sulfate-polyacrylamide gel electrophoresis
TOF: time-of-flight.
